# Prediction of seizure control in non-ketotic hyperglycemic induced seizures

**DOI:** 10.1186/1471-2377-9-61

**Published:** 2009-12-14

**Authors:** Somsak Tiamkao, Chitranon Janon, Kittisak Sawanyawisuth, Thongchai Pratipanawatr, Suthipun Jitpimolmard

**Affiliations:** 1 Department of Medicine, Faculty of Medicine, Khon Kaen University, 40002, Thailand

## Abstract

**Background:**

To study the factors predictive for seizure control in non-ketotic hyperglycemic induced seizures (NKHS).

**Methods:**

We studied 21 patients who were clinically diagnosed as NKHS at Khon Kaen University hospital, Thailand. Multiple linear regression analysis was used to identify the factors predictive for seizure control.

**Results:**

Most patients had no previous history of diabetes and presented with repetitive partial seizures. The mean number of seizure attacks was 45 times prior to admission. The average duration to terminate seizure was 36 hours and significantly predicted by frequency of seizures (estimate 0.9, p value 0.013).

**Conclusion:**

Frequency of seizures is the only predictive factor for the success of seizure control in NKHS.

## Background

Focal seizures can arise for various reasons; including non-ketotic hyperglycemic seizures (NKHS). NKHS has been firstly reported by James et al in 1969 [1] and defined as seizures concurrent with hyperglycemia without any other apparent causes such as brain infarction or sepsis [2-4]. Tiamkao et al have purposed the diagnostic clinical criteria in 2003 [5]. The cut-off point for plasma glucose and plasma osmolarity were more than 290 mg% and 288 mOsm/kg, respectively.

The common presenting symptom of NKHS is repetitive focal seizures. Recognition and awareness of this condition are crucial because the diagnosis is a guide for management of seizures. NKHS is usually resistant to anticonvulsant therapy. Phenytoin may even aggravate seizures [6]. Recently, there is no treatment guideline for this condition. Either subcutaneous or intravenous insulin without anti-epileptic medication was shown to be effective [5]. Even though NKHS is not a fatal condition or causes unconsciousness, it is repetitive, bothersome, and hard to control. This investigation studied the factors predictive for seizure control in NKHS. Research has not found that this issue has been previously reported in the literature.

## Methods

### Study population

A retrospective study was done at Srinagarind hospital, Khon Kaen University, Thailand. We searched the hospital database by using keywords seizure/seizures, epilepsy, convulsion, hyperglycemia, diabetes mellitus, and hyperglycemic seizure for all admitted patients between January 1, 1993 and December 31, 2003. Only patients with an age of more than 15 years old and met with our criteria were studied.

The clinical criteria for NKHS [5] were seizures presenting with plasma glucose more than 290 mg/dL, serum osmolarity more than 288 mOsm/L, seizure stopped after blood sugar and serum osmolarity declined below the level mentioned above, and the negative study of brain computed tomography (CT) scan.

According to the previously reported diagnostic criteria [2-4], cases were excluded if they had a seizure concurrent with sepsis, septic shock, central nervous system infection, septic encephalopathy, or other metabolic encephalopathy such as uremic encephalopathy, hepatic encephalopathy, hypo/hyperphosphatemia, hypo/hypercalcemia, hypo/hypermagnesemia and hypoglycemia.

We collected data from patient records including age, sex, history of diabetes mellitus, type and location of seizure, time from seizure onset to admission, frequency of seizure attacks, initial plasma glucose and serum osmolarity, and time to seizure control. Type of seizure was clinically defined as partial or generalized seizure. Frequency of seizure attacks were applied for one who had repetitive seizures and defined by total numbers of seizure clusters prior to admission. Time to seizure control was defined by duration between treatment and seizure termination.

### Statistical analysis

Descriptive statistics were used to show the patients' baseline characteristics. Covariates were fitted to find the correlation with time to seizure control by univariate and multivariate linear regression analysis. The final multiple linear regression model was identified by using the best subset selection model method with the lowest Akaike's information criterion value.

### Ethical approval

The study protocol was approved by the institutional review board and the ethics committee of Khon Kaen University.

## Results

There were 54 potential patients in the hospital database during the mentioned period; only 21 patients were met with the criteria. The other 33 patients were excluded by various exclusion criteria (15 patients had no brain imaging, 11 patients had abnormal brain imaging; ten patients had cerebral infarction and one patient had encephalomalacia, 6 patients had plasma glucose less than 290 mg/dL or serum osmolarity less than 288 mOsm/L, and 1 patient was sepsis).

Out of 21 patients, 11 were male. The median age was 61 year old (range 34-83). Thirteen patients (61.9%) had no previous history of diabetes and none of them had epilepsy as an underlying disease. Partial seizure was the most common presenting seizure type in 17 patients (epilepsia partialis continua in 16 patients and complex partial seizure in 1 patient). The other four patients had generalized tonic clonic seizure. The most common site of partial seizure was the left hand or left upper extremity (11 patients).

In average, the patients had seizure attacks 45 times prior to admission and took 5 days to reach hospital in a range 6-336 hours. The mean initial plasma glucose and serum osmolarity was 532.9 mg/dL and 302.1 mOsm/L. The mean (S.D.) duration to seizure control was 36 (36) hours; data available in 15 patients. The mean (S.D.) post-ictal plasma glucose was 190.3 (110.4) mg/dL, range 20-436.

**Figure 1:**
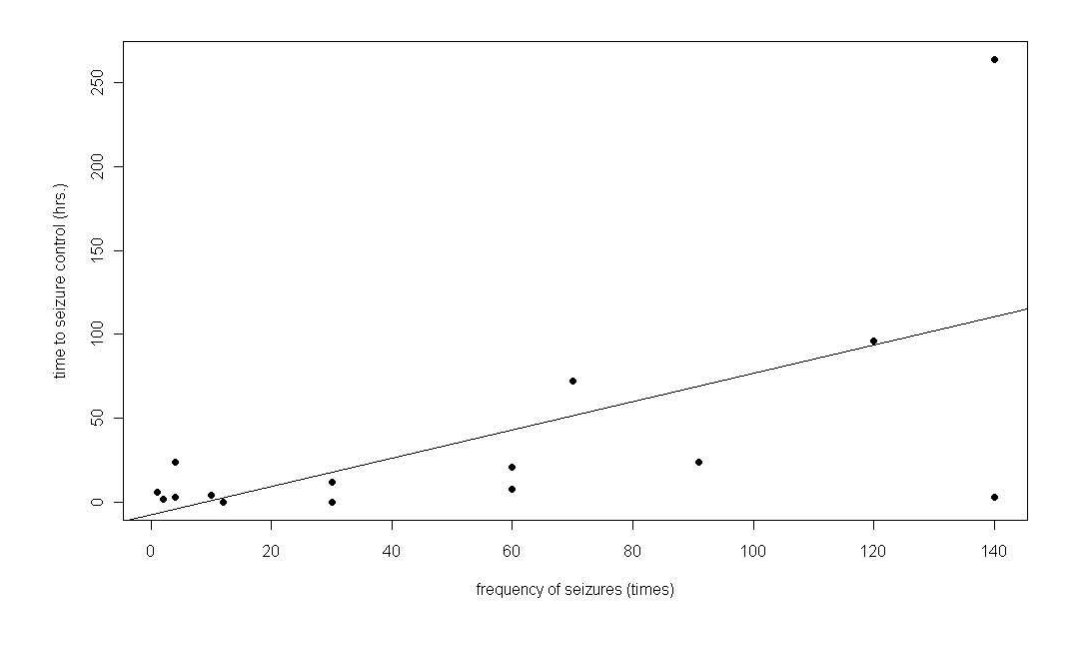
**The correlation between time to seizure control from 15 cases and frequency of seizures**.

The significant factors that were correlated to time to seizure control by univariate linear regression analysis were time prior to admission and frequency of seizure attacks (Table 1). In the final model of multivariate linear regression, frequency of seizures was the only significant factor related to time to seizure control (estimate 0.9, p value 0.013). The correlation between time to seizure control and time to admission was shown in figure 1.

**Table 1:** Baseline covariates and estimate value for time to seizure control by univariate linear regression analysis in patients with non-ketotic hyperglycemic induced seizures.

Variables	Mean (S.D.)	Estimate	p value
Age (years)	61 (34-83)*	0.7	0.728
Male gender (N)	11	53.8	0.137
Type of seizure			
EPC (N)	16	1	
GTC (N)	4	-27.58	0.6171
Time prior to admission (hr)	96 (6-336)*	0.2	0.127
Frequency of seizures (times)	44.9 (52.4)	0.8	0.013
Site of body having seizure			
Left side (N)	11	1	
Right side (N)	5	-12.8	0.778
Both sides (N)	1	-31.5	0.594
Initial plasma glucose (mg/dL)	532.9 (164.9)	-0.02	0.841
Initial serum osmolarity (mOsm/L)	302.1 (8.7)	-1.2	0.633

Note: EPC; epilepsia partialis continua, GTC; generalized tonic clonic seizure, * show median (range), N; number.

All patients received no prior treatment. The seizure episodes were initially controlled with subcutaneous regular insulin or continuous administration then later changed to oral hypoglycemic agents or subcutaneous insulin. All of the seizures were successfully controlled by strict control of blood sugar. One patient developed hypoglycemia during insulin therapy. None the patients needed long-term antiepileptic medication and no mortality was detected. After five years follow-up, none of the patients developed NKHS again.

## Discussion

NKHS is a rare diabetic complication. The most common presenting symptom is repetitive seizures caused by severe hyperglycemia. Similar to hyperglycemic hyperosmolar state, an emergency diabetic complication, NKHS patients have severe hyperglycemia with hyperosmolarity. There are, however, some different features.

Hyperglycemic hyperosmolar state may deteriorate patients' consciousness [7] and usually occurs in established diabetic patients. In contrast, more than half of our NKHS patients (61.9%) had never been diagnosed as having diabetes mellitus and those with partial seizure attacks retained their consciousness. Some certain precipitating factors such as serious infection or cardiovascular events evoke hyperglycemic hyperosmolar state. NKHS in our series, on the other hand, happened in naïve diabetic patients without any obvious precipitating factors. If, however, NKHS is disregarded by patients or diagnosis is missed or delayed, the episode might turn to hyperglycemic hyperosmolar state.

Seizures as the presenting symptom of NKHS are intermittent, repetitive and bothersome. Even with insulin treatment, the mean duration to terminate seizures was 36 hours. The longest time to seizure control was 264 hours or 11 days. NKHS mostly attacks the left upper arm or hands in 64.7% of patients without disturbance of the consciousness level. For these reasons, most patients seek healthcare later than other seizure conditions.

From the present data, frequency of seizures is significantly related to time to seizure control. The estimate value of 0.9 indicated that the addition of one seizure attack needed one more hour to control seizures. Intermittent focal seizures may be explained by a focal transient abnormal focal area in the brain [8,9] due to hyperosmolarity as in hyperglycemic hyperosmolar state. Gamma amino butyric acid, which has been shown to elevate the seizure threshold, may be low in nonketotic coma [10].

When compared to our previous report [5], the overall clinical manifestations are very similar in such parameters as percentage of partial seizure (95.6 vs. 80.9), affected body part (both a predominance of the left upper extremity), percentage of treatment-naïve diabetes mellitus patients (71.4 vs. 61.9), mean duration of seizure (5 vs. 4.9 days), and initial plasma glucose range 290-1099 vs. 299-979). The first number represents previous study.

Previously, physicians in this hospital usually treated NKHS with insulin therapy without having a CT scan of the brain [5]. The results from this study, included only subjects with the negative CT scan of the brain, showed comparable clinical manifestations and outcomes. Therefore, insulin treatment can be initiated without brain imaging of a limited of CT brain, particularly in Thailand if clinical manifestations are compatible with our criteria. If, however, seizures are still uncontrolled even with the plasma glucose levels less than 200 mg/dL or in elderly patients, the request for brain imaging is justified. Lowering the plasma glucose usually controls NKHS and no antiepileptic agent is needed [5,11,12].

There were some limitations in our study. Due to the retrospective design, some information might be lost. A potential recall bias in our study was the total numbers of seizure clusters prior to admission. However, we do believe that the patients should be able to give the approximate numbers of seizure clusters because the seizure attacks occurred intermittently and the patients were conscious during the focal seizure attacks. Sepsis is known as a contributing factor to high plasma glucose and may lead to seizure attack. We excluded patients with sepsis due to the previously purposed diagnostic criteria [2-4]. However, only one patient with sepsis was excluded in our study.

## Conclusion

Even though most seizure attacks in NKHS are partial seizures and do not cause unconsciousness; they are repetitive, bothersome and sometimes difficult to control. A higher frequency of seizures prior to treatment is an important predictive factor for the success of seizure control. Clinical recognition and prompt insulin therapy are crucial for a satisfactory outcome in NKHS.

## Competing interests

The authors declare that they have no competing interests.

## Authors' contributions

ST and SJ participated in the design of the study, interpreted data, and drafted the manuscript. CJ and TP collected and verified data. KS analyzed and interpreted data. All authors read and approved the final manuscript.

## Pre-publication history

The pre-publication history for this paper can be accessed here:

http://www.biomedcentral.com/1471-2377/9/61/prepub
